# A multicentre cohort study assessing the utility of routine blood tests as adjuncts to identify complete responders in rectal cancer following neoadjuvant chemoradiotherapy

**DOI:** 10.1007/s00384-022-04103-z

**Published:** 2022-03-24

**Authors:** Eastern Rectal Cancer Response Collaborative, Ireland, John Armstrong, Ishwarya Balasubramanian, Ann Brannigan, Ronan Cahill, Fiachra Cooke, Ben Creavin, Christina Fleming, Gerard McVey, Helen Mohan, Jurgen Mulsow, Conor Reid, Éanna Ryan, Conor Shields, Karl Schmidt, Kieran Sheahan, Des Winter

**Affiliations:** 1 Department of Academic Surgery, Cork University Hospital, Cork, Ireland; 2 Department of Radiation Oncology, St. Luke´s Radiation Oncology Centre, Dublin, Ireland; 3 Department of Colorectal Surgery, University Hospital Waterford, Waterford, Ireland; 4 Department of Colorectal Surgery, Mater Misericordiae University Hospital, Dublin, 7 Ireland; 5 Department of Colorectal Surgery, St Vincents University Hospital, Dublin, 4 Ireland; 6 Department of Pathology, St Vincents University Hospital, Dublin, 4 Ireland

**Keywords:** Rectal cancer, Neoadjuvant chemoradiotherapy, MRI, Neutrophil–lymphocyte ratio, Pathological response

## Abstract

**Purpose:**

Management of rectal cancer with a complete clinical response (cCR) to neoadjuvant chemoradiotherapy (NACRT) is controversial. Some advocate “watch and wait” programmes and organ-preserving surgery. Central to these strategies is the ability to accurately preoperatively distinguish cCR from residual disease (RD). We sought to identify if post-NACRT (preoperative) inflammatory markers act as an adjunct to MRI and endoscopy findings for distinguishing cCR from RD in rectal cancer.

**Methods:**

Patients from three specialist rectal cancer centres were screened for inclusion (2010–2015). For inclusion, patients were required to have completed NACRT, had a post-NACRT MRI (to assess mrTRG) and proceeded to total mesorectal excision (TME). Endoluminal response was assessed on endoscopy at 6–8 weeks post-NACRT. Pathological response to therapy was calculated using a three-point tumour regression grade system (TRG1-3). Neutrophil–lymphocyte ratio (NLR), platelet-lymphocyte ratio (PLR), serum albumin (SAL), CEA and CA19-9 levels post-NACRT (preoperatively) were recorded. Variables were compared between those who had RD on post-operative pathology and those with ypCR. Statistical analysis was performed using SPSS (version 21).

**Results:**

Six hundred forty-six patients were screened, of which 422 were suitable for inclusion. A cCR rate of 25.5% (*n* = 123) was observed. Sixty patients who achieved cCR were excluded from final analysis as they underwent organ-preserving surgery (local excision) leaving 63 ypCR patients compared to 359 with RD. On multivariate analysis, combining cCR on MRI and endoscopy with NLR < 5 demonstrated the greatest odds of ypCR on final histological assessment [OR 6.503 (1.594–11.652]) *p* < 0.001]. This method had the best diagnostic accuracy (AUC = 0.962 95% CI 0.936–0.987), compared to MRI (AUC = 0.711 95% CI 0.650–0.773) or endoscopy (AUC = 0.857 95% CI 0.811–0.902) alone or used together (AUC = 0.926 95% CI 0.892–0.961).

**Conclusion:**

Combining post-NACRT inflammatory markers with restaging MRI and endoscopy findings adds another avenue to aid distinguishing RD from cCR in rectal cancer.

## Introduction

Up to 30% of patients who undergo neoadjuvant chemoradiation (NACRT) for management of locally advanced rectal cancer (LARC) have the potential to achieve a complete pathological response [1–3]. Achieving a complete clinical response (cCR) and ultimately a complete pathological response (ypCR) is associated with an improved 5-year disease-specific and overall survival (90% and 87%, respectively) compared to those that do not [4]. It has also been shown that achieving cCR and ultimately ypCR is associated with less local recurrence (OR 0.25, 95% CI 0.10–0.59) and less distant failure (OR 0.23 95% CI 0.11–0.47) when compared to other groups [5]. A “watch and wait” approach has been proposed in patients that achieve cCR as an alternative to extensive surgical resection [6–8]. The advantages of such an approach are the obvious avoidance of surgical mortality and morbidity particularly relating to anorectal, urinary and sexual dysfunction and in elderly co-morbid patients [9–11]. Patients who undergo surgical resection compared to those managed by “watch and wait” report worse Wexner incontinence scores and higher daily defecation frequency [12]. Greater than two-thirds report urinary urgency and/or incontinence, and many require long-term urinary catheterisation (indwelling urinary catheter or intermittent self-catheterisation) [13]. Almost 75% of men who undergo pelvic resection for rectal cancer can report some element of erectile dysfunction [14]. Long-term stoma complications are also reported [15].

The greatest obstacle to the “watch and wait” approach is the difficulty in consistent, accurate identification of complete clinical responders (cCR) preoperatively [16]. Currently the most common method of assessment of response to NACRT is pelvic MRI with endoscopy; however, it is accepted that there is no single best test for identifying cCR as both modalities have inherent limitations [17, 18]. While the specificity of rectal MRI for establishing circumferential resection margin (CRM) involvement is high, its ability to differentiate between cCR and RD is less reliable [19]. Other imaging modalities, e.g., PET imaging has been suggested as superior to MRI for identifying cCR and RD but is still not consistently precise and has limitations [20].

The use of inflammatory markers as biomarkers in cancer is gaining interest, and in this setting, they may further improve the identification of cCR compared to RD following NACRT. Tumour hypoxia and necrosis following local and systemic therapies render a pro-inflammatory state post-NACRT [21, 22]. Therefore, it is expected that tumour response should correlate with systemic markers of inflammation. We hypothesise that raised inflammatory markers in the post-NACRT period reflects good tumour response to NACRT due to this correlation between tumour necrosis and inflammation. Thus, we expect that higher levels of inflammatory markers are observed in the setting of cCR compared to lower levels when less response is observed and residual disease remains. The aim of this study was to identify if inflammatory marker levels combined with post-NACRT MRI and endoscopy findings can improve the diagnostic accuracy for differentiating cCR from RD in rectal cancer following NACRT using complete pathological response as the reference standard.

## Methods

This multi-centre retrospective cohort study was performed across three tertiary referral rectal cancer centres in Ireland. A prospective database of all rectal cancer patients is maintained in each centre. Patients attending between 2010 and 2015 (inclusive) were assessed for inclusion. As regulated by the National Cancer Control Programme (NCCP), all rectal cancer cases are discussed at weekly multidisciplinary team (MDT) meetings in each centre comprising specialists in colorectal surgery, radiology, pathology and medical and radiation oncology where consensus management decisions are made based on best practice guidelines [23]. For the purpose of this study, the following inclusion criteria applied: diagnosis of histologically confirmed locally advanced rectal cancer; standard long course neoadjuvant chemoradiation (NACRT) was administered and completed; post-NACRT MRI was performed (mrTRG assessed); total mesorectal excision (TME) was performed to allow for complete histological analysis of the tumour bed and lymph nodes and definitive diagnosis of ypCR compared to evidence of RD. Due to the variability in endoscopy practice, this was not essential for inclusion but was analysed where available.

### Neoadjuvant chemoradiotherapy and timing of MRI

NACRT regimes typically consisted of 50–54 Gray (Gy) external beam radiation delivered in daily fractions over 5 consecutive weeks. A combination of IMRT or 3D conformal radiation methods was utilised depending on tumour location and patient factors. Concurrent administration of fluorouracil (5-FU) and folinic acid was also administered for radiosensitisation of the tumour bed. 5-FU-based capecitabine oral twice daily dosing was preferred. As part of pre-NACRT staging, all patients underwent MRI pelvis with included patients undergoing restaging MRI 6–8 weeks post-NACRT completion (preoperative).

### MRI and endoscopic assessment of response

A clinical response TNM stage (ycTNM) was calculated for restaging on MRI using mrTRG grading system to perform standardised reporting of tumour and lymph node response on MRI, and both T2- and diffusion-weighted sequences were used to perform this [24, 25]. All MRI assessments were performed by specialised consultant radiologists with a special interest in gastrointestinal imaging. Lymph nodes were considered “positive” if either border irregularity or mixed signal intensity could be demonstrated [24]. Complete clinical response (cCR) was diagnosed when no residual tumour or nodal disease was evident on MRI imaging (TRG1). Restaging endoscopy was performed in a subset of included patients in a similar timeframe to restaging MRI scan (6–8 weeks following completion of NACRT). This was based on local hospital guidelines and/or individual surgeon preference. When endoscopy was performed post-NACRT, endoluminal response was objectively assessed by a senior colorectal surgeon. An endoscopic diagnosis of complete response was made if there was no residual tumour visible or only a small residual erythematous ulcer or scar .

### Inflammatory markers

Appendix outlines the inflammatory markers studied with laboratory reference ranges. All blood tests were performed and measured within 7 days prior to commencing NACRT and again 6 weeks post-NACRT in the preoperative period to allow for appropriate time interval for response to NACRT to occur. Albumin was used as an inverse predictor of poor prognostic outcome [26]. Albumin levels were analysed using a Roche Biochemistry Platform which employs a colorimetric assay (pH based). Full blood counts were analysed using the Sysmex XN 200 Analyser using a particle counting method based on size and density. In all laboratories, National External Quality Assessment Scheme (NEQAS) testing is performed in conjunction with daily internal control assessments to allow for accuracy of data and standardisation of results. Neutrophil–lymphocyte ratio (NLR) ≥ 5 was deemed “high”, as is previously validated [27]. Platelet-lymphocyte ratio (PLR) ≥ 160 was classified as raised, a cut-off previously used for prognosis prediction in gastrointestinal malignancies [28].

### Histopathology assessment

Following surgical excision, specimen was fixed in formalin for histopathological assessment. Specimen analysis was performed in accordance with guidelines and protocols outlined by the American Joint Committee on Cancer, 7th Edition [29]. Tumour response to neoadjuvant therapy was calculated based on a three-point tumour regression grade system (TRG 1–3) whereby TRG 1 = complete response, TRG 2 = partial response and TRG 3 = no response [30]. Positive nodal status applies to > N0 nodal stage based on histological assessment. Circumferential resection margin (CRM) was classified as clear (R0) if > 1 mm of CRM was observed as tumour-free (R1 = tumour observed within 1 mm; R2 = CRM tumour positive).

### Statistical analysis

Statistical analysis was performed using SPSS, version 22. Patients were classified as ypCR or RD (defined by final histological assessment). Continuous variables were analysed using Mann–Whitney U and paired t-test with statistical significance observed at *p* < 0.05. Categorical variables were assessed using Fisher exact or χ2 test. Following dichotomisation of inflammatory variables (low and high) based on the median value [31], multivariate logistic regression analyses were performed to identify factors associated with ypCR. Factors were selected based on published literature and clinical knowledge and combined in a binomial logistic regression model. The accuracy of investigative tests for ultimate ypCR was calculated using diagnostic accuracy testing, e.g. sensitivity and specificity. Furthermore, area under the receiver-operating curve was calculated to assess model discrimination. Factors with greatest association with ypCR in the multivariate model were included in this analysis. Outcomes were dichotomised and a logistic regression approach utilised.

## Results

**Fig. 1:**
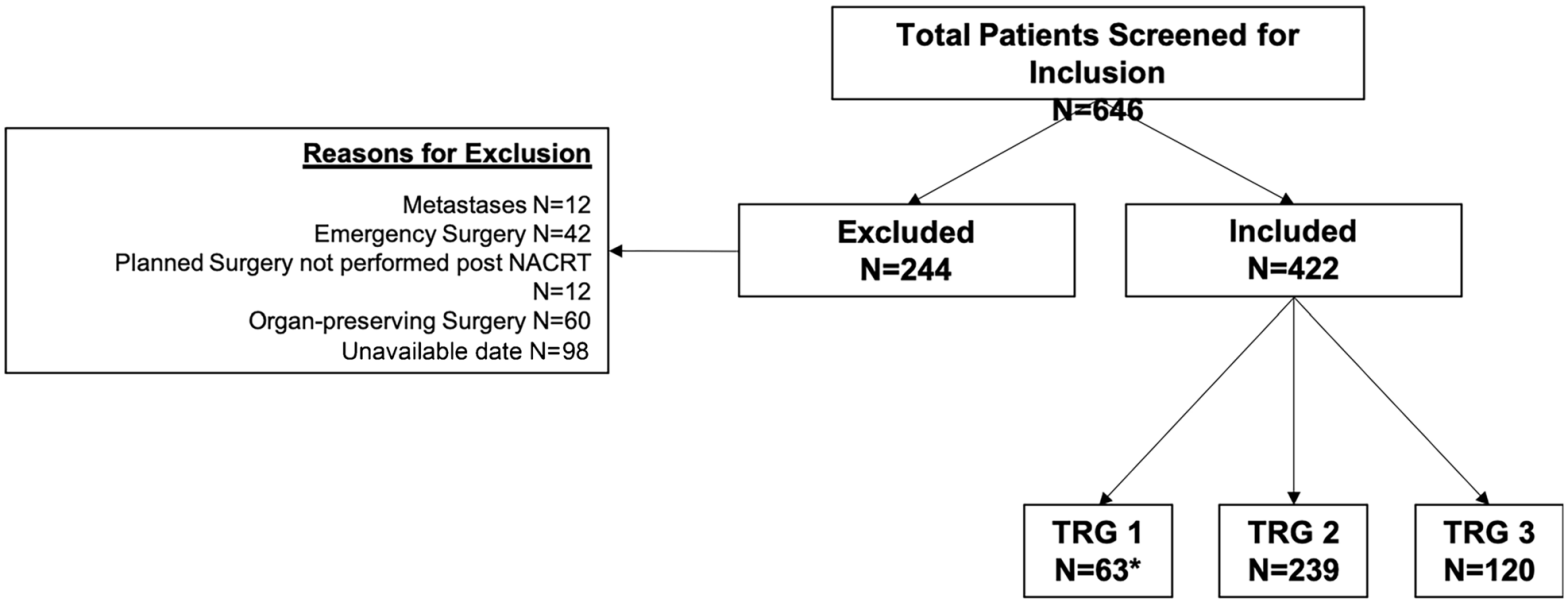
Patient selection for study inclusion. NACRT, neoadjuvant chemoradiotherapy. TRG, tumour regression grade. Single asterisk shows that complete pathological response for the entire cohort is 25.5% (this includes TRG 1 (*N* = 63) and those who proceeded for organ-preserving surgery (*N* = 60))

A total of 646 rectal cancer patients were screened for inclusion. Overall, the rate of cCR was 25.5% (*n* = 63 who were included in final analysis and *n* = 60 who were excluded from final analysis as organ-preserving surgery performed in the form of local excision). As outlined in Fig. 1, a total of 244 patients were excluded and 422 included in final analysis (*n* = 63 ypCR, *n* = 359 = RD). Table 1 shows that age (*p* = 0.194), tumour level (*p* = 0.241), pre-NACRT tumour stage (*p* = 0.169) and nodal status (*p* = 0.439) were comparable between those who achieved ypCR and those that did not. However, the ypCR group contained significantly more male patients (74.6% vs 62.7%, *p* = 0.044).

**Table 1:** Summary of patient and tumour characteristics with breakdown into patients with residual disease. Compared to those who achieved complete pathological response following neoadjuvant chemoradiotherapy

**Characteristics**	**Residual disease** **Non-ypCR** **[*N* (%)]** **(*n* = 359)**	**Complete response** **ypCR** **[*N* (%)]** **(*n* = 63)**	***p* value**
**Age (years)** Mean (SD) Range	64 (11.8) 30–90	66 (8.78) 46–85	0.194
**Gender** Male Female	225 [62.7] 134 [37.3]	47 [74.6] 16 [25.4]	0.044
**Tumour level** Proximal Mid Distal	60 [16.7] 134 [37.3] 165 [46]	12 [19] 28 [44.4] 23 [36.5]	0.241
**Pre-NACRT tumour stage **^**a**^ II III IV	32 [8.9] 293 [81.6] 34 [9.5]	9 [14.3] 50 [79.4] 4 [6.3]	0.169
**Pre-NACRT nodal status **^**a**^ Positive Negative	274 [76.3] 85 [23.7	47 [74.6] 16 [25.4]	0.439
**Post-NACRT MRI **^**b**^ Residual disease ycCR	343 [96.1] 14 [3.9]	23 [71.9] 9 [28.1]	<0.001
**Endoscopy **^**c**^ Residual disease Endoluminal complete response	19 [90.5] 1 [7.7]	2 [9.5] 12 [92.3]	<0.001

*ypCR* complete pathological response, *ycCR* complete clinical response, *NACRT* neoadjuvant chemoradiotherapy, *SD* standard deviation

^a^Pre-NACRT tumour and lymph node stage obtained on initial staging pelvic MRI

^b^Post-NACRT MRI available in 389 patients

^c^endoluminal assessment with endoscopy following NACRT available in 34 patients

### Restaging MRI and endoscopy

Of the 359 patients with RD on histology, the majority were T3 on initial MRI staging [81.6% (*n* = 293)] with a minority staged as T2 [8.9% (*n* = 32)] and T4 [9.5% (*n* = 34)]. Post-NACRT MRI was performed in 389 of included patients. A significant difference in T stage at presentation was not observed between those with RD and ypCR following NACRT (*p* = 0.241). Two-thirds of patients with RD were node positive at presentation [76.3% (*n* = 274)], and similar node-positive status was observed in the ypCR group [74.6% (*n* = 47), *p* = 0.439]. Restaging MRI was available in 389 patients (92%). On restaging MRI (post-NACRT), 96% (*n* = 343) of patients with RD on histology had definitive evidence of such RD on restaging MRI with the remaining 4% (*n* = 14) inaccurately identified as a cCR. The sensitivity of restaging MRI for identifying cCR was 64% (95% CI 0.54–0.88), and specificity was 92% (95% CI 0.76–0.97). This equates to a sensitivity of 96% [95% CI 94–98%] and specificity of 28% [95% CI 16–45%]. Endoscopy to assess endoluminal response was available on 34 patients. In this subgroup, endoscopy demonstrated greater accuracy than MRI for identifying cCR with a specificity of 93% (95% CI 0.80–0.97) and sensitivity of 84% (95% CI 0.71–0.99).

### Combining MRI, endoscopy and inflammatory markers to identify complete pathological response (ypCR)

Multivariate analysis findings are outlined in Table 2. Complete clinical response (cCR) on MRI [OR 2.121 [1.459–3.085] *p* = 0.002] and cCR on endoscopy [OR 2.251 [1.53–3.952] *p* = 0.022] demonstrated an increased odds of ultimate ypCR. This association was greater when MRI and endoscopy combined [OR 4.503 [2.349–8.556] *p* < 0.001] and when MRI and endoscopy findings were further combined with PLR > 160 [OR 4.618 [1.545–6.804] *p* < 0.001]. The greatest association was demonstrated when cCR findings on MRI were combined with cCR findings on endoscopy and NLR > 5 [OR 6.503 [1.594–11.652] *p* < 0.001].

**Table 2:** Multivariate logistic regression analysis to assess factors predictive of a final complete pathological response following NACRT using patient and tumour characteristics, inflammatory markers alone, MRI and endoscopic findings and combined inflammatory markers with endoscopic and MRI findings

	**Multivariate (logistic regression) analysis**
	***OR [95% CI] p value***
**Age**	0.926 [0.857–1.001] 0.286
**Gender**	1.620 [0.952–2.755] 0.068
**Tumour level**	0.926 [0.857–1.001] 0.241
**Tumour stage**	0.986 [0.896–1.085] 0.151
**Nodal status**	1.082 [0.642–1.822] 0.768
**MRI (cCR)**	**2.121 [1.459–3.085] 0.002**
**Endoscopy (cCR)**	**2.251 [1.53–3.952] 0.022**
**CEA**	1.414 [0.704–2.839] 0.448
**CA19-9**	1.047 [0.358–3.064] 0.158
**SAL**	1.073 [0.628–1.835] 0.173
**NLR > 5**	1.051 [0.655–1.688] 0.609
**PLR > 160**	1.506 [0.955–2.374] 0.168
**Endoscopy (cCR) + MRI (cCR)**	**4.503 [2.349–8.556] < 0.001**
**NLR > 5 + MRI (cCR)**	**3.573 1.710 [0.732–4.565] < 0.001**
**PLR > 160 + MRI (cCR)**	**1.350 [1.004–1.816] 0.003**
**Endoscopy (cCR) + MRI (cCR) + NLR > 5**	**6.503 [1.594–11.652] < 0.001**
**Endoscopy (cCR) + MRI (cCR) + PLR > 160**	**4.618 [1.545–6.804] < 0.001**

*OR* odds ratio, *cCR* complete clinical response, *CEA* carcinoembryonic antigen, *CA19-9* cancer antigen 19–9, *SAL* serum albumin level, *NLR* neutrophil–lymphocyte ratio, *PLR* platelet-lymphocyte ratio

### Diagnostic accuracy and test strength

**Fig. 2:**
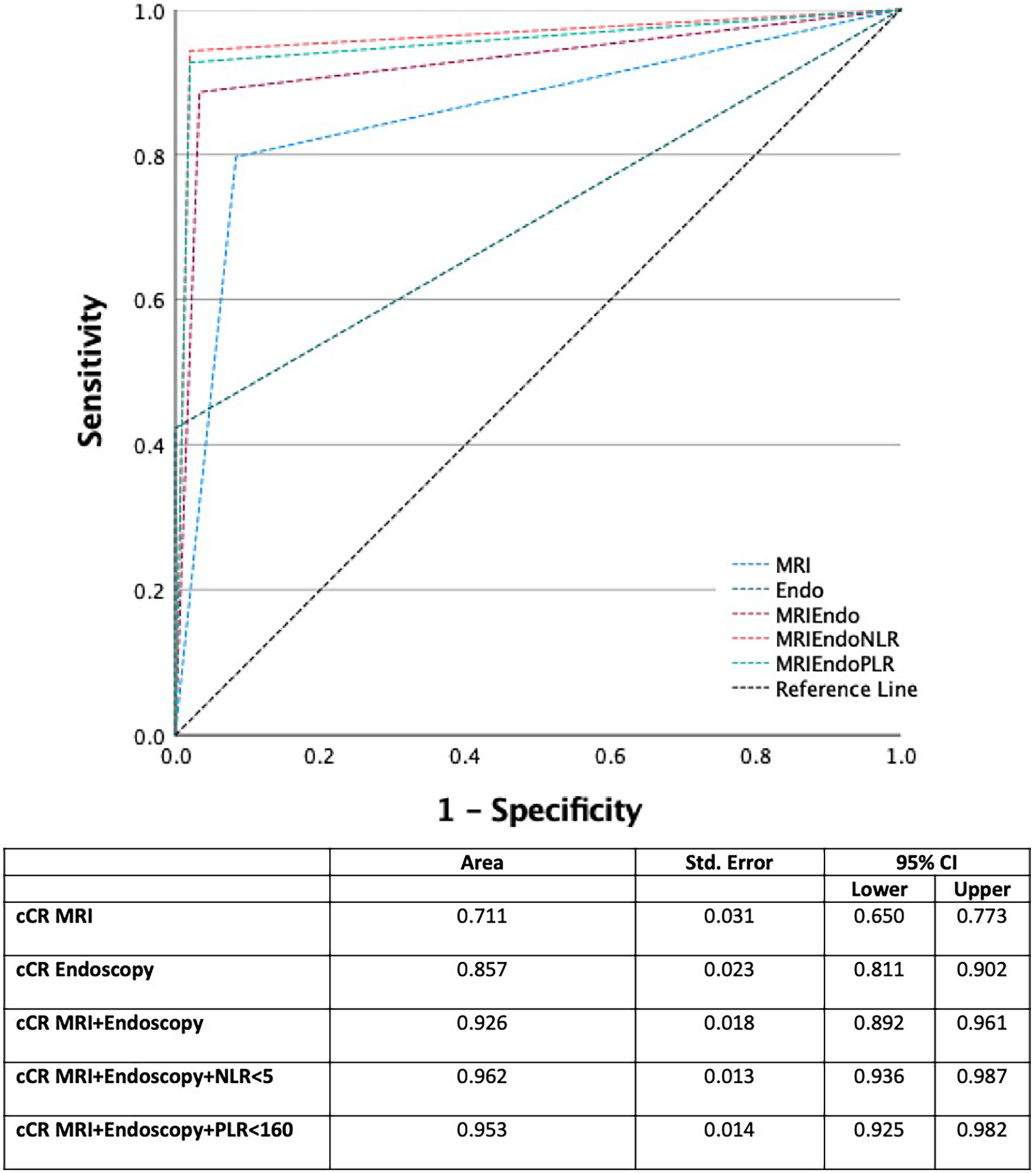
Receiver operating curve (ROC) representing the ability to predict complete pathological response (ypCR). cCR, complete clinical response. NLR, neutrophil–lymphocyte ratio. PLR, platelet-lymphocyte ratio

The diagnostic accuracy of the most significant diagnostic tests, alone and in combination, is summarised in Table 3. While MRI and endoscopy independently have high specificity for identifying cCR (92% and 93%, respectively), their sensitivity is less (64% and 84%, respectively). Using MRI and endoscopy together improves the sensitivity to 88%. Combining the findings of NLR < 5 post-NACRT with endoluminal evidence of cCR on endoscopy and cCR on post-NACRT MRI further improves the sensitivity to 92% while maintaining specificity at 97%. Addition of PLR < 160 does not improve the diagnostic accuracy any greater than MRI and endoscopy used together. The receiver operating curve (ROC) in Fig. 2 further confirms the superiority of combining NLR < 5 with cCR on MRI and endoscopy to post-NACRT MRI or endoscopy alone or in combination. The area under the curve (AUC) is highest for cCR on MRI and endoscopy combined with NLR < 5 (AUC = 0.962 95% CI 0.936–0.987) compared to MRI alone (AUC = 0.711 95% CI 0.650–0.773), endoscopy alone (AUC = 0.857 95% CI 0.811–0.902) or MRI combined with endoscopy (AUC = 0.926 95% CI 0.892–0.961).

**Table 3:** Diagnostic accuracy of restaging strategies for identifying ultimate complete pathological responders

	**Sensitivity** [95% CI]	**Specificity** [95% CI]	**PPV** [95% CI]	**NPV** [95% CI]
cCR MRI	0.64 [0.54–0.88]	0.92 [0.76–0.97]	0.73 [0.61–0.86]	0.93 [0.22–0.59]
cCR Endoscopy	0.84 [0.71–0.99]	0.93 [0.80–0.97]	0.84 [0.71–0.98]	0.94 [0.86–0.99]
cCR MRI + Endoscopy	0.88 [0.77–0.99]	0.95 [0.77–0.99]	0.88 [0.77–0.99]	0.96 [0.83–0.99]
cCR MRI + Endoscopy + NLR > 5	0.91 [0.79–0.99]	0.97 [0.78–1.0]	0.94 [0.84–1]	0.97 [0.71–0.99]
cCR MRI + Endoscopy + PLR > 160	0.92 [0.81–0.99]	0.98 [0.70–0.97]	0.92 [0.71–0.98]	0.97 [0.68–0.99]

*PPV* positive predictive value, *NPV* negative predictive value, *cCR* complete clinical response, *PLR* platelet-lymphocyte ratio, *NLR* neutrophil–lymphocyte ratio

*Restaging = investigations performed following completion of NACRT and prior to surgery

## Discussion

Organ preservation is gaining interest in rectal cancer as patients who achieve a complete clinical (cCR) or complete pathological response (ypCR) have superior oncological outcomes. The main obstacle to “watch and wait” management of rectal cancer in cCR is the lack of an indisputable method to distinguish cCR from residual disease (RD) following NACRT. We are therefore likely, in many cases over treating patients with TME for ypCR and the associated surgical morbidity risks [9–11]. To date no single test has a consistently high specificity and sensitivity for cCR following NACRT in rectal cancer. However, we have identified in this study that the combination of inflammatory markers, MRI and endoscopic findings is better than any test alone. The combination of NLR and MRI findings of mrTRG1 significantly increases the sensitivity of distinguishing cCR compared to RD preoperatively with combining PLR with MRI and endoscopic findings also demonstrating superiority. These methods may improve the ability to identify those who need to proceed to formal TME surgery compared to those who may benefit from “watch and wait” programmes or organ preserving surgery.

It is well established that achieving cCR or ypCR in rectal cancer is associated with superior oncological and survival outcomes [4, 5]. While this is an area of vast advancement over the past decade, obstacles still exist to definitively distinguishing patients who have cCR and RD without a full oncological resection and definitive pathological evidence [18, 20, 32]. It has been established that both timing and type of restaging modality are crucial [1, 33]. It has also been proposed that disease response to NACRT is an evolutionary event and may continue beyond the standard time that is observed in current practice [34]. This further raises the question of optimum timing for surgery in rectal cancer patients following NACRT. The “perfect” time for surgery has not been definitively established in keeping with the personalised evolutionary concept of “response” to NACRT. While the results of the GRECCAR-6 trial suggests that routinely waiting longer to operate (i.e., week 11 compared to week 7 post-NACRT) has no overall benefit, further evidence is emerging that this may not be the case and certainly it appears that waiting for a minimum of at least 8 weeks offers benefit [35, 36].

The modality of restaging is a topic for much debate. MRI (standard T2 and diffusion-weighted sequences) is currently the most widely used method for restaging rectal cancer following NACRT combined with endoscopy. MRI accuracy however can be suboptimal [37]. While MRI has greater sensitivity and specificity (77% and 94%, respectively) for identifying mesorectal fascial involvement with tumour (circumferential resection margin (CRM) in total mesorectal excision), its ability to identify complete pathological response can still be inconsistent [38, 39]. More recently MRI with a surface phased-array coil has been proposed as the preferred imaging modality for evaluation response to NACRT [37]. On meta-analysis, 18F-FDG PET scanning has shown good potential for diagnosing ypCR (pooled sensitivity and specificity of 71% and 76%, respectively) [40]. Of concern, it has been reported that a false-negative rate as high as 17% for nodal disease may occur on restaging of LARC patients with PET [40–42]. Accuracy of endoscopic assessment for evidence of endoluminal features of tumour response has been reported as > 90% [43]. In this study, we have identified that combining inflammatory marker levels can improve the diagnostic accuracy of restaging MRI and endoscopy to distinguish cCR from RD, with NLR > 5 combined with cCR on restaging MRI and endoscopy most significant. These findings suggest that exploring the role of inflammatory markers in restaging strategies in rectal cancer could be further explored.

It is established that raised systemic inflammatory markers prior to commencing NACRT is associated with more aggressive disease and poorer prognostic outcomes and survival in colon and rectal cancer [25, 44–46]. We chose in this work to investigate the usefulness of systemic inflammatory markers post-NACRT for rectal cancer. We hypothesised that inflammation in this setting correlates with response, and the greater the inflammatory response, the greater the chance of cCR. A number of studies support this hypothesis. Firstly, tumour hypoxia and necrosis are proposed to stimulate a systemic inflammatory response following administration of NACRT [47]. It has further been shown that an anti-tumour immune response may occur secondary to the process of tumour cell death following administration of ionising external beam radiation [22, 48]. From this, it may be postulated that the degree of tumour response to NACRT should correlate with the inflammatory response stimulated. It has been confirmed that the number of dying cells in response to treatment can reach a threshold where sufficient signals are stimulated to in turn stimulate antigen-presenting cells to activate a systemic immune or inflammatory response [48]. It has also been shown in mouse models of breast cancer that local radiotherapy can stimulate a systemic immunological response to allow a more systemic management in poorly immunogenic metastatic tumour cells [48]. With this particular work in mind, it has been suggested that a complete pathological response to NACRT could represent evidence for successful in situ and systemic immunisation against tumour, a concept supported by its survival benefit compared to other magnitudes of response [48]. Based on this evidence and our findings, there are grounds to accept that raised inflammatory markers following NACRT correlates with disease response to therapy due to the release of immunological markers during the process of cell death and tumour necrosis which manifests as an increase in systemic inflammatory marker levels.

There are a number of limitations to this study. This is a retrospective study with the associated limitations. Sufficient data for 98 patients was unavailable, and thus they were excluded from final analysis. Post-NACRT MRI results were “inconclusive” in 7.8% (*n* = 33) and could not distinguish definitive cCR from RD; therefore, these were removed from final analysis. Clinical assessment with endoscopy is important following NACRT, in particular for surveillance programmes and “watch and wait”. Endoscopy may have a particular role in those with inconclusive findings on restaging MRI. In this study, post-NACRT endoluminal assessment with endoscopy was available in less than 10% of patients. The practice of performing post-NACRT endoscopy is expanding over the past number of years, and the small number performed in our cohort may be due to the study period but also surgeon preference.

In conclusion, the role of post-NACRT inflammatory markers combined with MRI and endoscopy findings to distinguish ypCR from RD in rectal cancer is a promising area for further research and exploration. This is particularly true in the expanding era of “watch and wait” management and proposed organ-preserving surgery. These tests are routinely performed, inexpensive and readily available for all patients. This adds another avenue to aid distinguishing RD from ypCR identification in rectal cancer as an adjunct to MRI and endoscopy.

## Appendix

Normal laboratory reference values used. *CEA* carcinoembryonic antigen, *CA19-9* cancer antigen 19–9.

**Table:** 

**Biomarker**	**Reference range**
CEA	*0.0–5.2ug/L*
CA19-9	*0.6–25 U/ml*
White cell count	*4–10* × *10*^*9*^*/L*
Lymphocyte count	*1–3* × *10*^*9*^*/L*
Neutrophil count	*2–7* × *10*^*9*^*/L*
Platelet count	*150–400* × *10*^*9*^*/L*
Serum albumin level	*35–50 g/L*

## Data Availability

Data is available by reasonable request from the corresponding author.
